# Neighborhoods and Aging: How Place-Based Assets and Challenges Shape Mental Health and Well-Being

**DOI:** 10.1093/geroni/igaf122.2117

**Published:** 2025-12-31

**Authors:** Megan Nizza, Clara Scher

## Abstract

Neighborhood environments play a critical role in shaping the mental health and well-being of older adults, particularly those with intersecting minoritized identities such as race, cognitive status, and socioeconomic position. This symposium examines how neighborhood-based assets and barriers contribute to disparities in later-life health, emphasizing both protective (e.g., neighborhood cohesion and accessible infrastructure) and adverse factors (e.g., poor housing conditions and experiences of discrimination). The first presentation explores how neighborhood contexts support and challenge the health and well-being of older adults racialized as Black and living with mild cognitive impairment or dementia. The second presentation examines geographic and age-related variations in experiences of discrimination among self-identified Black women, discussing the extent to which neighborhood racial composition affects the reporting frequency of discrimination. The third presentation examines how perceived neighborhood disorder and cohesion are associated with later-life cognitive function, underscoring the mediating role of psychological resilience. The fourth presentation investigates how public housing quality and broader neighborhood contexts interact with age to shape mental health outcomes, offering implications for housing interventions to mitigate disparities. Finally, the fifth presentation explores neighborhood-level facilitators and barriers to social connectedness for older people living with cognitive impairment or mild dementia, proposing community-driven solutions to neighborhood designs. Through this interdisciplinary lens, presenters will conclude with a discussion on research, practice, and policy implications, emphasizing the need for neighborhood-level innovations to improve the mental and psychosocial well-being of older adults with intersectional social positions.

